# Case Report: Sirolimus as management strategy for thrombocytopenia related to ARPC1B deficiency

**DOI:** 10.3389/fimmu.2026.1845298

**Published:** 2026-08-04

**Authors:** Gianluca Dell’Orso, Martina Guardigni, Elena Palmisani, Enrico Drago, Federica Penco, Ignazia Prigione, Giuseppina Conteduca, Lucia Augusta Baselli, Martina Rossano, Damiano Lemmi, Maria Carla Giarratana, Eugenia Mariani, Luca Arcuri, Stefano Volpi, Maurizio Miano

**Affiliations:** 1 Hematology Unit, IRCCS Istituto Giannina Gaslini, Genoa, Italy; 2 Department of Neurology, Rehabilitation, Ophthalmology, Genetics, Maternal and Child Health (DINOGMI), Università degli Studi di Genova, Genoa, Italy; 3 Clinical and Experimental Immunology, IRCCS Istituto Giannina Gaslini, Genoa, Italy; 4 Gene Therapy Program, Boston Children’s Hospital, Harvard Medical School, Boston, MA, United States; 5 Rheumatology and Autoinflammatory Diseases Unit, IRCCS Istituto Giannina Gaslini, Genoa, Italy; 6 Laboratory of Human Genetics, IRCCS Istituto Giannina Gaslini, Genoa, Italy; 7 Pediatric Immunorheumatology Unit, Fondazione IRCCS Ca’ Granda Ospedale Maggiore Policlinico, Milan, Italy

**Keywords:** actin cytoskeleton, ARPC1B deficiency, inborn error of immunity (IEI), sirolimus, thrombocytopenia

## Abstract

Biallelic mutations in ARPC1B gene are responsible for an inborn error of immunity (IEI) characterized by thrombocytopenia and combined immunodeficiency with heterogeneous immune-dysregulatory features. Sirolimus is a mammalian target of rapamycin (mTOR) inhibitor, which is successfully used in patients affected by primary or secondary autoimmune cytopenias. We report the case of a patient suffering from refractory/relapsing thrombocytopenia secondary to a previously unreported homozygous variant on ARPC1B and successfully managed with sirolimus. Additional mild immune dysregulatory features, such as lymphoproliferation and autoantibodies, also resolved after sirolimus treatment. Protein’s functional analysis showed an interaction between ARPC1B’s role in actin cytoskeleton function and mTOR signaling pathway. However, the actin polymerization assay evaluation on samples collected before and after sirolimus treatment showed no significant differences. This result might suggest that the efficacy of sirolimus in controlling thrombocytopenia was mainly due to its immunomodulatory effect to reduce autoimmunity. The absence of a direct effect on actin cytoskeleton may also explain the lack of efficacy of sirolimus in previously reported ARPC1B-deficient patients affected by broader immune dysregulation features, including at least three or more systems involved. Although increased evidence is supporting early hematopoietic stem cell transplantation (HSCT) as an option for more severe broad-spectrum phenotypes, sirolimus could represent a therapeutic option in ARPC1B deficiency in case of a less severe immune-dysregulation phenotype or as a bridge therapy before transplant. Prospective observation can help in defining patients’ evolution and prognosis with sirolimus monotherapy, and the risks and benefits of combination strategies with steroids or immune-modulating drugs should be compared with HSCT progress and results in transplant-related mortality.

## Introduction

Genetic defects impairing actin cytoskeleton function may lead to broad phenotypic consequences. Wiskott–Aldrich syndrome (WAS) and ARPC1B deficiency are both classified as immunodeficiency with congenital thrombocytopenia in Table 1 of the Human Inborn Errors of Immunity classification (1). WASP protein, encoded by *WAS* gene (2), interacts with the Arp2/3 complex, leading to the binding of actin monomers and daughter filament growth (3). The ARP2/3 complex is composed of actin-related proteins ARP2 and ARP3, associated with ARPC2, ARPC3, ARPC4, ARPC5, and ARPC1, a WD40 repeat-containing protein (4–7). ARPC1A and ARPC1B are two isoforms of ARPC1 in humans (4), located in tandem on human chromosome 7 (8) and characterized by a 68% amino acid sequence homology (7). ARPC1B expression is restricted to hematopoietic cells (7), playing a key role in the development of adaptive immune cells and thrombocytes (9). Biallelic mutations in *ARPC1B* cause ARPC1B deficiency, an autosomal recessive disorder presenting with thrombocytopenia and combined immunodeficiency (7). Immunological dysfunction leads to frequent ear, skin, and lung infections, increased levels of immunoglobulin E (IgE) and A (IgA), positive autoantibodies (ANA and ANCA) and altered lymphocyte subsets with low CD3+ T cells and increased B cells (mainly transitional) (7, 10, 11), severe eczema, food allergies, asthma, adenopathies, and gastrointestinal hemorrhages (12). Treatment options reported in nearly 40 patients range between the use of immunosuppressors to manage immune dysregulation features to hematopoietic stem cell transplantation (HSCT) (7, 9–19). Sirolimus has been reported as a treatment strategy in combination with steroids or other immunosuppressants in 10 patients (12, 20) affected by a broad spectrum of immune dysregulatory features, but no specific reports on its functional role in improving the pathogenesis of the disease have been published so far. Herein we report the case of a child born from non-consanguineous parents carrying an unreported homozygous intronic deletion on the *ARPC1B* gene (c.64 + 4_64 + 24DEL) and suffering from isolated severe thrombocytopenia. His specific mild clinical phenotype proved responsive to sirolimus treatment.

## Case report

A 7-month-old boy of Latin-American ancestry born to non-consanguineous parents was admitted to a hospital in El Salvador due to immune thrombocytopenia (ITP). No relevant disorders were reported in the family history and in his neonatal period. He was initially treated with intravenous immunoglobulins (IvIg, 2 g/kg) and high-dose methylprednisolone, followed by prednisone (2 mg/kg/day), which was slowly tapered over 5 months due to steroid dependency. In the following months, after moving to Italy, he received several administrations of IvIg at 1 g/kg every 21 days and later at 400 mg/kg/day for 5 days. The high-dose intravenous methylprednisolone led to a transient partial response (PLT 60 × 10^9^/L). The immunological screening and lymphocyte subsets at first evaluation in our center showed a shift toward activated CD3+ HLA DR+ cells and reduced CD3+CD4+CD25br+CD45RA- T regulatory cells. Additionally, a reduction of CD3+ T cells was associated to a relative increase in CD19+ B lymphocytes, but no significant alterations in absolute counts of T, B, and NK cells according to age were found (**Table 1**). Abdominal ultrasound showed an enlarged liver related to hepatic steatosis and increased splenic diameter above the upper normal limit for his age. Due to refractory cytopenia, bone marrow evaluation with trephine biopsy was performed, and it showed a slightly reduced marrow cellularity with a quantitative reduction of megakaryocytes, an increased percentage of eosinophils, and mixed B and T cell infiltration. A chest CT scan was performed, and it showed lymphoproliferative features with subcentimetric lymph nodes in the right para-tracheal region and bilateral axillary regions. The Immune Deficiency and Dysregulation Activity (IDDA) 2.1 score was 15 (21).

**Table 1:** Lymphocyte subsets and immunological screening.

	Baseline (1 year old)	Reference values for age	During treatment (3 years old)	Reference values for age
	uL (%) or % alone	/uL (%) or % alone	/uL (%) or % alone	/uL (%) or % alone
Lymphocytes	4,430 (55.4)	2,700–11,900 (51–69)	6,250 (61.5)	1700-6,900 (53–77)
CD3+ T cells	2,140 (48)	1,400–8,000 (53–71)	2,820 (45)	900–4,500 (56–77)
CD3+ CD4+ T helper cells	1,460 (33)	900–5,500 (34–46)	1,250 (27.7)	500–2,400 (32–45)
CD3+ CD8+ T cytotoxic cells	550 (12.6)	400–2,300 (15–22)	810 (13)	300–1,600 (19–25)
CD3+ HLADR+ T activated cells	500 (11.5)	100–700 (5–7)	120 (3.4)	80–400 (5–7)
CD19+ B cells	1,940 (43.8)	600–3,100 (20-27)	1,760 (28.2)	200–2,100 (18–24)
CD16+CD56+CD3- NK cells	230 (5.2)	100–1,400 (8–11)	1,470 (23.5)	100–1,000 (7-9)
CD3+CD4+/CD3+CD8+ ratio	2.6	1.7–2.5	2	1.3–1.9
CD3+CD4+CD25br+CD45RA- T regulatory cells	0.2	0.2–0.4	0.5	0.5–0.7
CD3+TCRαβ+	45.2	45–61	41.2	50–68
CD3+ TCRγδ+	2.9	3.3-4.5	3	5–7
CD3+TCRαβ+CD4-CD8-	0.7	<1.7	0.8	<1.7
B220+	48.8	<60	9	<60
CD19+CD27+	4.6	>15	6.7	>15
CD3+CD25+/CD3+HLADR+ ratio	0.2	>1	0.3	>1
CD27-CD10++CD38++ transitional B cells	7.8	8–16	4.4	5–10
CD27-CD10+-CD38+-IgD+ naive B cells	84.4	80–90	84.9	75–85
CD27+IgD+IgM+ marginal zone B cells	4	1.5–4	3.5	4–9
CD27+IgD-IgM- switched memory B cells	1.6	<2	1	1–7
CD27+IgD-IgM+ pre-switched memory B cells	0.1	<1	0.9	<1
CD27+IgD+IgM- IgD memory	0.1	<0.5	1.1	<0.5
CD27-CD10-CD38++ pre-plasmablasts	1.6	n.a.	1.3	n.a.
CD27++CD21-CD38++ plasmablasts	0.7	n.a.	0	n.a.
CD21lowCD38low	10.3	<5	15.1	<5
CD27-IgD- double negative B cells	4	<10	8	<10
CD27-CD21-CD21low double negative 2 B cells	1.1	<25	45.3	<25
CD27-CD10+-CD38+-IgD+CD21low activated naïve B cells	10.9	<5	29.76	<5
Anti-nuclear antibodies	1:80		Negative	
Anti-platelet antibodies	Positive		Negative	
Direct/indirect antiglobulin test	Negative/positive		Negative/negative	
Anti-thyroid peroxidase	Positive (normal thyroid function)		Negative	
Immunoglobulin G	3,179 mg/dL (increased due to recent IVIg infusions)		1,050 mg/dL	
Immunoglobulin A	70 mg/dL		217,mg/dL	
Immunoglobulin M	43 mg/dL		99,mg/dL	
Immunoglobulin E	312 kU/L		102 kU/L	
IDDA 2.1 score	15		4.5	

Bold cell entries refers to values out of reference values for age.

**Figure 1:**
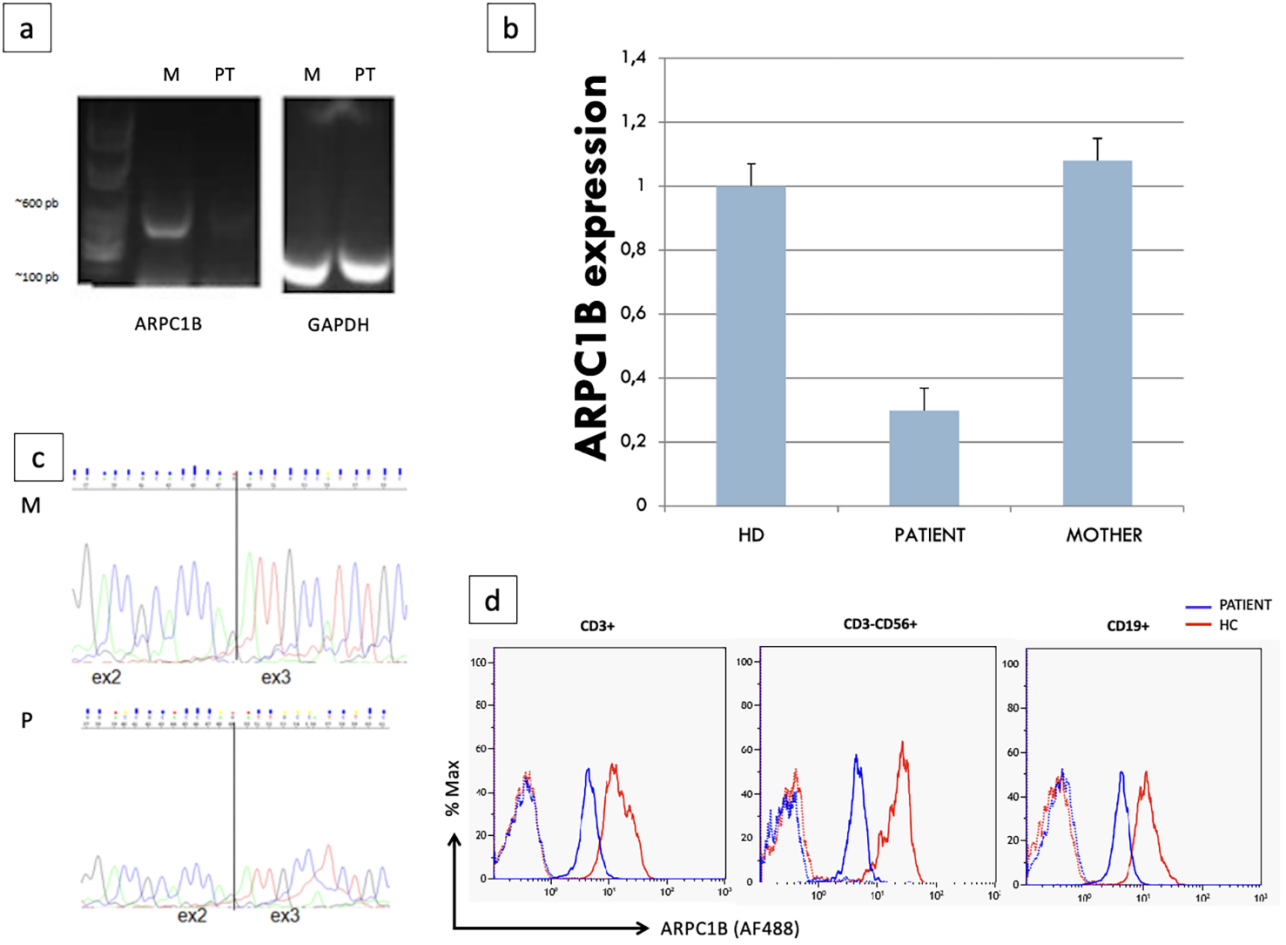
**(a)** Agarose gel showing the PCR-amplified cDNA expression of ARPC1B. **(b)** *ARCP1B* qPCR expression related to a healthy donor. **(c)** Sanger sequences of ARPC1B cDNA. **(d)** Flow cytometry analysis of ARPC1B expression in patient T, NK, and B circulating lymphocytes. Representative histograms of flow cytometry analysis for ARPC1B in T (CD3+), NK (CD3-CD56+), and B (CD19+) lymphocytes from the patient (blue) and a healthy control subject (HC) (red). Dotted lines represent isotype control. HD, healthy donor; M, mother; Pt, ARPC1B patient.

A next-generation sequencing (NGS) gene panel analysis focused on 407 genes related to IEIs showed a previously unreported homozygous intronic deletion on *ARPC1B* gene (c.64 + 4_64 + 24DEL). This variant was classified as of uncertain significance according to its extremely low frequency in the general population (22). Complementary DNA (cDNA) analysis revealed reduced *ARPC1B* gene expression by means of reverse transcriptase PCR product analysis and quantitative PCR (**Figures 1a, b**). cDNA sequencing revealed only the wild-type allele (**Figure 1c**), suggesting nonsense-mediated mRNA decay or the inability of the designed PCR primer to amplify the mutant allele. A cytofluorimetric analysis of ARPC1B protein showed a reduced expression in T, B, and NK lymphocytes compared to a healthy control (**Figure 1d**). Familial genetic analysis showed a heterozygous state for *ARPC1B* variant in both parents.

**Figure 2:**
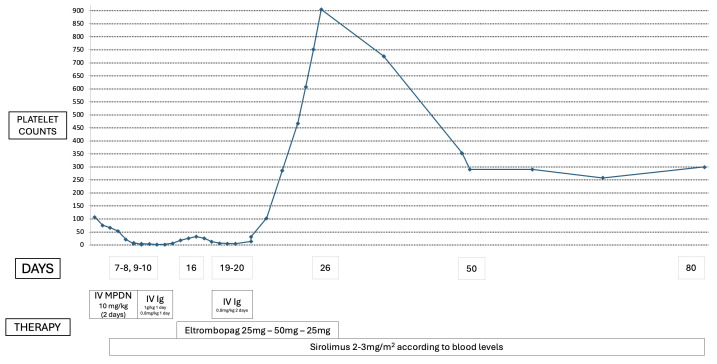
Platelet counts and treatment during the first 80 days of follow-up at our center.

**Figure 3:**
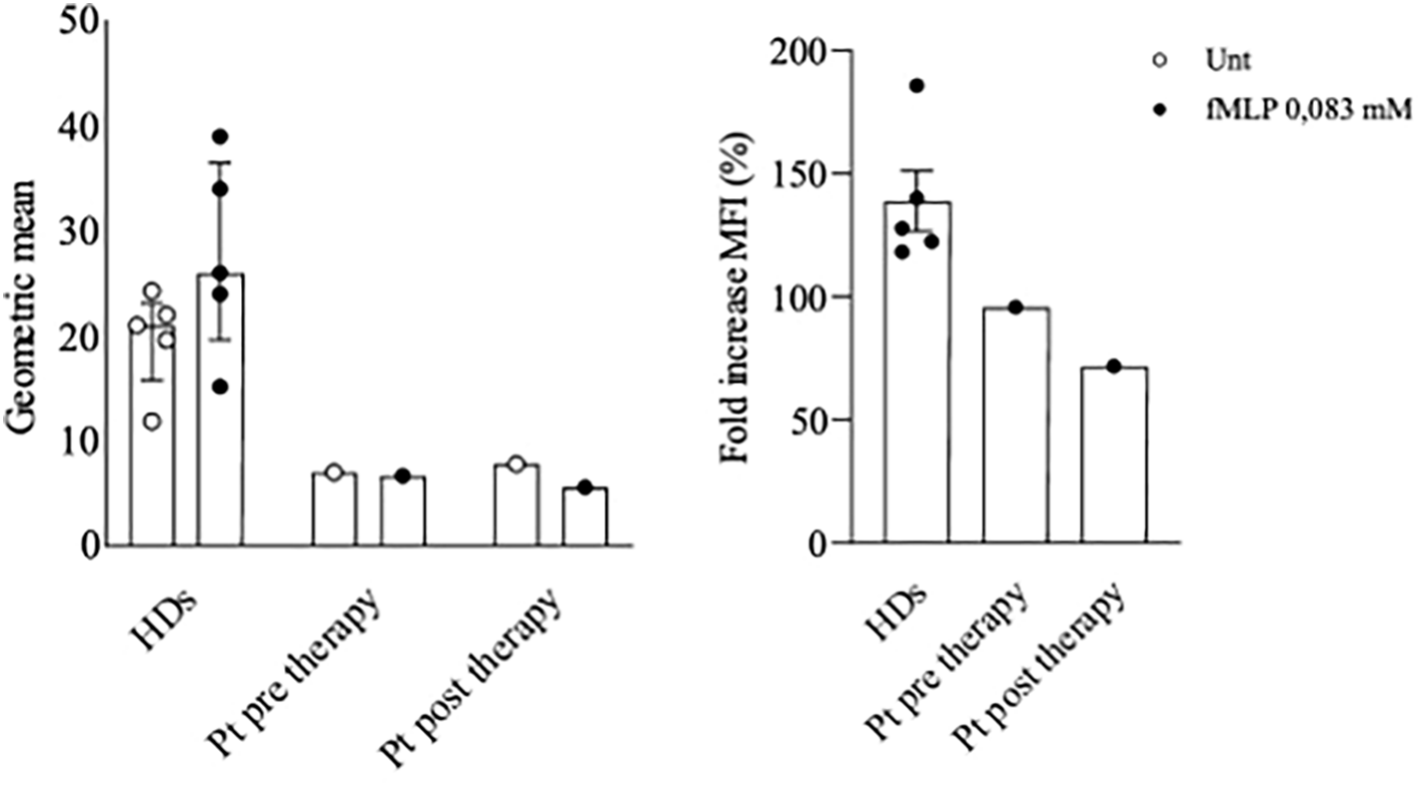
Actin polymerization in the patient pre- and post-therapy compared with those of healthy donors. CD14 monocytes untreated or treated with fMLP showing the polymerization of actin as an increase of the geometric mean of Alexa Flour 647-Phalloidin staining after stimulation **(A)**. Increase of actin polymerization is shown as fold increase in MFI-stimulated samples/MFI-unstimulated samples (×100) **(B)**.

Due to the poor response to IvIg and steroid dependency, sirolimus was started at a dose of 2 mg/m^2^/day. During the first 2 weeks, no response was noted (**Figure 2**), but the combination of TPO agonist eltrombopag at 25 mg/day with sirolimus at 3 mg/m^2^/day led to a fast increase in platelet levels of up to above 750 × 10^9^/L. Therefore, TPO agonist was weaned after only 10 days of treatment, but the laboratory response was not lost. The platelet counts reached stable values between 150 × 10^9^/L and 300 × 10^9^/L with single-agent sirolimus (target serum levels 7–15 ug/mL). At 3.5 years of follow-up, the platelet counts are stable in the normal reference range, and the lymphocyte subsets and immunological profile showed a significant improvement (**Table 1**) compared to baseline, with an associated improvement in CD3+CD4+CD25br+CD45RA- T regulatory cell fraction. The abdominal ultrasound and CT scan demonstrated a reduction in lymphoproliferative features, with normal hepatic and splenic size and residual subcentimetric lymph nodes only in the left axillary region. The IDDA 2.1 score improved to 4.51 (21). In order to investigate a potential direct effect of sirolimus on the cytoskeleton, an actin polymerization assay was performed in samples collected before and during sirolimus treatment, and no differences were observed (**Figure 3**).

## Discussion

Steroids and other supportive strategies to manage severe immune dysregulation have been reported (7, 9–19) in APRC1B deficiency, but increasing evidence nowadays is supporting early HSCT as an option for more severe broad-spectrum phenotypes (23). Immunosuppressors are mentioned as a treatment in combination strategies or as steroid-sparing agents (12, 20), but specific reports on efficacy are lacking due to the difficulty in describing a specific response in multisystemic autoimmunity. In particular, sirolimus has been reported in 10 patients from two case series (12, 20), but to the best of our knowledge, this is the first report on its use for a specific clinical phenotype in APRC1B deficiency.

The patient was referred to our center due to isolated refractory ITP. Despite a very mild immune dysregulatory phenotype with no vasculitis, colitis, eczema, or relevant infections, the patient received the diagnosis of ARP1B deficiency thanks to the finding of a homozygous intronic deletion on *ARPC1B* gene (c.64 + 4_64 + 24DEL), and a reduced ARPC1B expression was demonstrated through molecular and cytofluorimetric analysis (**Figure 1**). Overall, 53 cases carrying 27 different genetic variants, mostly homozygous and less frequently compound heterozygous, have been reported so far (**Table 2**). Four variants (c.64C>T, C.64+1G>A, c.64+1G>C, and c.64+2T>A) are known to be located in the same splice region (10, 12, 16, 20, 24) as the new one identified in our patient. Protein expression data in literature are limited and conflicting, mainly due to the limited number of cases. In the case series by Volpi et al. (12), variants c.64 + 1G>C and c.64 + 2T>A were associated with undetectable levels of ARPC1B protein, and the pathogenic hypothesis was related to altered splicing. However, structural modeling for c.64 + 1G>C and c.64 + 1G>A variants has shown that the insertion of additional amino acids at the loop between the first two beta-strands could potentially lead to interference with protein folding or to the formation of a disulfide bridge, resulting in abnormal protein–protein interactions (10). In addition, a variable expression of ARPC1B in different cellular subpopulations has also been observed (10). In our case, protein expression was reduced to approximately 20% compared to that of a healthy donor, and reduction appeared similar in T-, B-, and NK subsets (**Figure 1**).

**Table 2:** ARPC1B variants reported in literature.

Number	Origin	*ARPC1B* genetic variant	Zygosity	Intron or exon	Amino acidic change	Ref.
2	Mexico	c.64C>T, c.899_944del	Compound heterozygous	Exon 2, exon 8	p.Gln22Ter, p.Glu300GlyfsTer31	(16)
1	Turkey	c.64+1G>A	Homozygous	Donor splice site of intron 2	NA	(10)
1	Italy	c.64+1G>C	Homozygous	Donor splice site of intron 2	NA	(10, 12, 23)
15	Nepal	c.64+2T>A	Homozygous	Donor splice site of intron 2	NA	(12, 24)
2	Nepal	c.64+2T>A, c.784-1G>A	Compound heterozygous	Donor splice site of intron 2, exon 7		(20)
1	Mexico	c.94G>A, c.111G>C	Compound heterozygous	Exon 3	p.Val32Met, p.Lys37Asn	(16)
1	Italy	c.212_226del	Homozygous	Exon 4	p.Gly71_Asn75del	(15)
1	Colombia	c.258G>A	Homozygous	Exon 4	p.Trp86Ter	(10)
4	Slovenia	c.265A>C	Homozygous	Exon 4	p.Thr89Pro	(14)
1	South Asia	c.269_270dupCT	Homozygous	Exon 4	p.Val91TrpfsTer30	(7, 12)
1	Morocco	c.311G>C	Homozygous	Exon 4	p.Trp104Ser	(12, 23)
2	Scotland	c.314C>T	Homozygous	Exon 4	p.Ala105Val	(7, 12)
1	Morocco	c.318del	Homozygous	Exon 4	p.Asn107ThrfsTer13	(10)
1	Somalia	c.392+2T>C	Homozygous	Donor splice site of intron 4	NA	(12, 23)
1	Morocco	c.491_495TCAAGdelCCTGCCCins	Homozygous	Exon 4	p.Phe164SerfsTer31	(12, 13, 23)
1	Italy	c.622G>T	Homozygous	Exon 6	p.Val208Phe	(10, 12, 23)
2	Jewish (consanguinity)	c.G623DEL-TC	Homozygous	Exon 6	p.Val208ValfsTer20	(9, 10, 23)
1	Jordania	c.708-1G>A	Homozygous	Splice site acceptor of exon 6	NA	(12)
3	Afghanistan	c.783G>A	Homozygous	Exon 7	p.(Ala261Ala)	(11, 23)
1	Mexico	c. c.863del	Homozygous	Exon 8	p.Pro288Leufs*9	(18)
2	Iran	c.897_910delCGAGCGCTTCCAGA	Homozygous	Exon 8	p.Glu300ProfsTer153	(12)
3	Mexico	c.899_944del	Homozygous	Exon 8	p.Glu300GlyfsTer31	(16)
1	Mexico					(18)
1	Mexico					(49)
1	Turkey	c.1081-5T>G	Homozygous	Intron 9	NA	(17)
1	Canada	c.1087dup	Homozygous	Exon 10	p.Glu363GlyfsTer95	(10, 23, 50)
1	Kenya	NA	NA	Splice site	NA	(51)

The pathogenesis of thrombocytopenia in WAS and ARPC1B deficiency has been related to reduced bone marrow megakaryocyte count, decreased proplatelet formation (7, 12) as megakaryocyte podosomes are dependent on the WASP-Arp2/3 axis (25–27), and reduced calcium-rich and ATP-rich platelet-dense granules (7, 28, 29). However, some reports described WAS patients undergoing splenectomy to correct the platelet count and size (30, 31), in agreement with the hypothesis of peripheral immune-mediated thrombocytopenia and immune-dysregulation. In ARPC1B deficiency, a T-cell proliferation defect has been demonstrated, suggesting that the impairment of cytoskeletal dynamics may cause impaired signaling through T-cell receptor (TCR) (10), with altered organization of the immune synapse. Moreover, a defective chemokine-induced migration may be secondary to the impaired intracellular cytoskeletal machinery (10). An *in vitro* analysis of CD8+ cells also showed an altered cytotoxic response, potentially responsible for severe acute and prolonged viral infections (9, 32). In B cells, an *in vitro* analysis demonstrated altered BCR clustering on the plasma membrane, resulting in increased spontaneous B cell tonic signaling (33). Functionally, this alteration has been related to increased numbers of circulating B cells (7, 12) and transitional B cells, with skewed activation in response to microbial-associated molecular patterns and potentially to autoantigens (33). Accordingly, in our patient, a baseline reduction in T CD4+ and CD8+ cells was found in association with a relative expansion of B cells and generation of autoantibodies and autoimmunity. As a limitation, basal lymphocyte subsets and the normal B cell maturation profile could have been altered by recent repeated steroid treatment.

The presence of immune-dysregulation in the setting of bone marrow impairment has been reported in a number of diseases (34–37) and often represents a diagnostic challenge for clinicians since, in some cases, autoimmune cytopenia can overlap with marrow failure. Nowadays, it is well known that a relevant number of patients referred for single- or multilineage marrow failure may have an underlying IEIs (38). This knowledge may be helpful to plan the right treatment taking into consideration the use of immunosuppressors.

Sirolimus is a mammalian target of rapamycin (mTOR) inhibitor, reported as a treatment of patients with autoimmune lymphoproliferative syndrome (ALPS) and other primary or secondary autoimmune cytopenias (39–41). mTOR signaling in immune function plays a role in enabling and modulating T cells’ response to cytokines, co-stimulatory molecules, and antigenic signals (42). In ITP, mTORC1 signaling pathway is highly enriched, with an increased expression of (43) phosphorylated mTOR and its substrates S6K and Akt (44), and a robust inhibition of mTORC1 by sirolimus led to the reduction in mTORC1 activity.

Based on the hypothesis that thrombocytopenia could be, at least in part, related to an autoimmune mechanism, sirolimus was administered as a steroid-sparing agent in a highly pretreated child, leading to a complete response. The response was achieved after 2 weeks, simultaneously with the starting of a trial with a TPO agonist and after two additional doses of IvIg. We consider sirolimus to be responsible for the resolution of this therapy-refractory thrombocytopenia because the associated TPO agonist was weaned off in only 10 days to avoid an excessive increase in platelet count, and no rebound of thrombocytopenia was observed after such a short course of treatment. Additionally, other mild immune dysregulation features such as reduced T regulatory cells, lymphoproliferation, and autoantibodies showed a progressive improvement over time. Lastly, the IDDA score—a tool proposed to quantify the severity and widespreadness of an immune dysregulation disorder—supported an immunomodulatory effect of sirolimus, describing a shift to milder disease activity.

It is hard to speculate about the specific relationship between genotype and phenotype or response to sirolimus due to scarcity of specific data. Overall, sirolimus has been reported in the literature in 10 cases. According to the specific genetic defect, nine of 10 patients were diagnosed with a variant in the same intronic region as our patient. As summarized in **Table 3**, clinical phenotypes in treated patients were significantly more severe, with broader involvement of at least three systems (skin, bowel, and hematologic), a clearly more severe immune dysregulation and lymphoproliferative features, and a more relevant infectious history (12, 20, 23). In five patients, it was interrupted due to incomplete response, leading to HSCT (four patients) or death (one patient, after insufficient compliance to multiple medications). Overall, sirolimus is mentioned as a first-line steroid-sparing agent in only two patients. Most patients received multiple immunomodulating approaches after steroids. In three patients, an improvement in clinical condition has been reported, but no specific details on the clinical response in different systems or long-term follow-up data are available (**Table 3**).

**Table 3:** Reports of patients affected by ARPC1B deficiency and treated with sirolimus.

ARPC1B variant	Age at onset or referral	Clinical features	Laboratory features	Indication to sirolimus	Evaluation of response
c.622G>T p.Val208Phe (12)	First months	MAS, chronic CMV infection, failure to thrive, persistent hepatosplenomegaly, recurrent pustular skin lesions, recurrent infections; progressive lymphadenopathy (2 years); acute fever, parotiditis, painful abdominal wall lesion with macrophage muscular infiltrate on biopsy (4 years)	Lymphocytopenia, increased double negative T CD4-CD8- cells, intermittent thrombocytopenia	Steroid-sparing agent in the acute setting	Bridge to CD19/TCRαβ-depleted HSCT
c.64 + 1G>C (12)	1 month	Growth failure, eczema, recurring episodes of hemorrhagic enterocolitis, leukocytoclastic vasculitis; staphylococcal right upper lobe pneumonia, with residual pneumatocele; warts	Intermittent and later symptomatic thrombocytopenia; mild T-cell lymphopenia, low IgG/IgM, elevated IgE/IgA, eosinophilia	Steroid-sparing agent	Bridge to CD19/TCRαβ- depleted HSCT
c.64 + 2T>A (20)	5 years	Growth failure, recurrent infections (pneumonia, otitis, abscesses), bleeding episodes, eczema, vasculitic rash, lymphadenopathy, colitis	Thrombocytopenia, anemia, elevated acute-phase reactants, increased IgE/IgA, reduced IgM/IgG, increased B cells, reduced T CD4+ T, borderline low CD8+ cells, ANA + 1:160	Switch from mycophenolate mofetil as steroid-sparing agent	Relapsing course, indication to HSCT
c.64 + 2T>A (20)	2.5 years	Growth failure, recurrent infections, chronic diarrhea, eczema, vasculitic rash, lymphadenopathy, bilateral otomastoiditis, pansinusitis	Anemia, elevated acute-phase reactants, hypocalcemia, ANA + 1:80, reduced T CD4+ and CD8+ cells, increased B and NK cells, borderline anti-PM-Scl, elevated IgE/IgA and reduced IgM/IgG	Combination in multiple immune modulating strategy	Suboptimal response, indication to HSCT
c.64 + 2T>A (20)	Onset in infancy, referral at 8 years of age	Failure to thrive, skin rash, frequent pyogenic infections, polyarthritis and musculoskeletal symptoms; acute onset of leukocytoclastic vasculitis, evolving in acute intestinal obstruction, pneumonia, uveoretinitis	Anemia, thrombocytopenia, elevated acute-phase reactants, increased IgE/IgA, reduced IgG/IgM, ANA + 1:80, positive RF and ACPA (anti-cyclic citrullinated peptide antibody); reduced T CD8+ and naïve T CD4+ cells, increased NK cells	Combination in multiple immune modulating strategy	Not evaluable due to poor compliance to medications
c.64 + 2T>A (20)	Late referral at 14 years	Failure to thrive, recurrent pneumonia, bronchiectasis, cavitating lung lesions, skin infections, gastroenteritis, eczema, splenomegaly, renal abscess, hydronephrosis	Anemia, thrombocytopenia, eosinophilia, elevated acute-phase reactants; positive RF; increased IgE/IgA, reduced IgM/IgG. Low T CD3+ and CD8+ cells, naïve CD4+ T-cells	Tapering steroids, sirolimus, mycophenolate mofetil	Improvement in hematological and cutaneous involvement, ongoing follow-up for multisystemic involvement
c.64 + 2T>A (20)	15 months	Bloody diarrhea, recurrent respiratory and skin infections, severe eczema, failure to thrive, joint pain; late referral at 9 years of age: gross wasting, stunting, clubbing, vasculitic skin rash, knee and ankle arthritis, eczema, cavitation in lungs, bronchiectasis, hepatosplenomegaly, skin nodules; hypothyroidism and low growth hormone levels; eosinophilic colitis	Anemia, thrombocytopenia, elevated acute-phase reactants, increased IgE/IgA levels, reduced IgG/IgM. ANA + 1:160, positive RF; reduced CD8+ T-cells, increased NK cells	Combined immune modulation strategy with mycophenolate mofetil	Good clinical conditions reported
c.64 + 2T>A (20)	1.5 years old	Hematochezia, recurrent otitis and pneumonia, recurrent fever, eczematous skin lesions, irritability, failure to thrive	Anemia, thrombocytopenia, mild leukocytosis, raised inflammatory markers, increased IgE/IgA/IgG/IgM levels; reduced CD3+ T-cells, normal CD4+ T-cells, elevated CD19+ B-cells and CD56+ NK cells	Sirolimus as steroid-sparing agent	Mild improvement of symptoms
c.64 + 2T>A (20)	7 years old (retrospective diagnosis)	Eczema, nodular skin rash, cutaneous vasculitis, failure to thrive, history of recurrent otitis and pneumonia, chronic arthritis, mesenteric lymphadenopathy and hepatomegaly	Anemia, elevated inflammatory markers, increased IgE levels, ANA + 1:80, RF	Steroid-sparing agent	NA
c.64 + 2T>A (20)	7 months of age	Recurrent febrile episodes and respiratory infections (pneumonia, empyema, pleural effusion), arthritis, otitis media as well as externa, leukocytoclastic vasculitis, stunted growth, multiple skin manifestations like papulo-nodular lesions, pustules, atopic dermatitis, seborrheic dermatitis, healed hyperpigmented skin lesions in limbs, mild hepatosplenomegaly, myositis, and arthralgia	Anemia, leukocytosis, few records of thrombocytopenia, increased inflammatory marker, RF, IgE/IgA	Combined immune modulation strategy with steroid and mycophenolate mofetil	NA

ANA, antinuclear antibodies; CMV, cytomegalovirus; HSCT, hematopoietic stem cell transplantation; Ig, immunoglobulin; MAS, macrophage activation syndrome; ACPA, anti-cyclic citrullinated peptide antibody; NK, natural killer; RF, rheumatoid factor.

More recently, Quach et al. (24) proposed a hypothesis to explain the phenotypic differences between two patients from different families despite sharing an identical germline homozygous *ARPC1B* splice-site variant, ARPC1B c.64 + 2T>. Molecular studies suggested that the downstream cryptic splice-site activated by the mutation permits “leaky splicing”, enabling trace expression of wild-type ARPC1B and leading to a less severe phenotype in one case due to the differential transcriptional activity of wild-type ARPC1B. Further analysis is required to determine whether a similar mechanism could be involved in these patients, leading to differential protein expression among different lymphocyte subsets.

Since biological data in an experimental setting showed an interaction between ARPC1B’s role in actin cytoskeleton function and mTOR signaling pathway (45), we evaluated the actin polymerization assay on samples collected before and after sirolimus treatment, which showed no differences (**Figure 1**). This result might suggest that the efficacy of sirolimus in controlling thrombocytopenia was not due to the normalization of the actin defect but, rather, only to an immunomodulatory effect on immune dysregulation. The improvement of autoimmune markers and IDDA score and the increase in T regulatory cells after sirolimus treatment strengthen this hypothesis. The lack of efficacy of sirolimus in previously reported ARPC1B-deficient patients (12, 20) with a more severe phenotype can be related to the lack of effect on the specific defective mechanism.

This report suggests that the use of sirolimus in ARPC1B deficiency could represent an useful therapeutic option to manage less severe immune-dysregulation-related complications of the disease, but not to correct the actin cytoskeleton dysfunction. Additional investigations about the relationship between mTOR signaling pathway and actin cytoskeleton dysfuncton are required. Accordingly, in our patient, the disease management with sirolimus is potentially lifelong due to the progressive nature of ARPC1B deficiency. It is hard to predict long-term control in preventing broader clinical evolution, which would suggest a change in therapeutic strategy and an indication for HSCT. Therefore, careful monitoring of late-onset complications such as severe pulmonary vasculitis, malignancy, or refractory inflammatory bowel disease (IBD) should be included in the follow-up.

In addition, as infections have been reported after sirolimus introduction in patients with ARPC1B deficiency (12, 20), these events should be carefully monitored. Consistent with the conclusions of a retrospective analysis by Comella et al. (46), it is reasonable to consider the increased risk of infection in other ARPC1B-deficient patients treated with sirolimus (12, 20) as related to more severe underlying immune dysregulation phenotypes; no severe infectious events have been reported during a 35-month follow-up in our patient’s clinical history, which was characterized by a milder clinical phenotype and a more limited immune dysregulation phenotype. Additionally, in our patient, an increase in T CD8+ lymphocytes could reflect an improved defense to infections during sirolimus treatment.

This report shows that sirolimus may represent an alternative option for the management of immune thrombocytopenia secondary to immune dysregulation related to ARPC1B deficiency, and it may be proposed for limited clinical phenotypes since the proposal of HSCT must consider the related risks and benefits. Prospective observation can help in defining patients’ evolution and prognosis with sirolimus monotherapy or combination therapies. The risks and benefits of combination strategies with steroids or immune-modulating drugs should be compared with HSCT progress and results in transplant-related mortality. In this regard, a collaborative study by ESID-EBMT-CIS is ongoing to compare the clinical outcomes and quality of life of patients with ARPC1B deficiency managed either conservatively or with HSCT (47). Specific genotype–phenotype relationships (20) will be the focus of future studies to contribute to tailored patient treatment selection, as current data are not sufficient to identify genetic and molecular predictors of the clinical phenotype or response to treatment.

## Methods

### Reverse transcription, quantitative PCR, and cDNA sequencing

*ARPC1B* (GenBank accession no. BC007555) gene expression was analyzed by PCR and real-time PCR as previously described (48). Briefly, TRIzol reagent was used for RNA extraction (Thermo Fisher Scientific, MA, USA). We verified RNA quality using a NanoDrop ND-1000 and an Agilent 2100 Bioanalyzer. Synthesis of cDNA was carried out from 400 ng of total RNA according to the manufacturer’s protocol using the Advantage RT cDNA Kit (Clontech, Mountain View, CA, USA), followed by RNase H digestion. The PCR amplification program included a single denaturation step at 94 °C for 3 min, which was followed by 35 cycles at 94 °C for 1 min, then 62 °C for 1 min, and 72 °C for 2 min and a final extension step at 72 °C for 10 min. The following sense and antisense oligonucleotide pairs, respectively, were used: *ARPC1B* 5′-CAAGGACCGCACCCAGATT-3′ and 5′-TGCCGCAGGTCACAATACG-3′; *GAPDH*, 5′-GAGCAACAGGAAGTGGCTGTG-3′ and 5′-TAATGCTTCCAGTTTACAAGTGGT-3′. Quantitative real-time PCR was performed using LightCycler 480 SYBR Green I Master (Roche Diagnostics, Mannheim, Germany). *ARPC1B* expression was normalized to *GAPDH* using the 2−ΔΔCt method (48). Each experiment was performed in triplicate. The PCR products were then analyzed by electrophoresis on a 2% agarose gel stained with GelRed dye (Life Technologies, Carlsbad, CA, USA) and purified using QIAquick Gel Extraction Kit (QIAGEN). Sequence analysis was conducted using the ABI BigDye Terminator Ready Reaction Mix (Applied Biosystems, Foster City, CA, USA) and an ABI 3130XL Genetic Analyzer (Applied Biosystems, Foster City, CA) according to the manufacturer’s protocol.

### Flow cytometry for ARPC1B

Cells from whole blood were stained with CD3-APC, CD56-PE, and CD19-PeCy7 mAbs (Sony Biotechnology) for 15 min at room temperature. After RBC lysis (FACS Lysing Solution, BD Biosciences), the cells were fixed and permeabilized with Cytofix/CytoPerm™ solution (BD Biosciences) according to the manufacturer’s instructions. The cells were stained with rabbit polyclonal anti-ARP2/3 subunit 1 antibody (1:50; ab99314 abcam) or rabbit IgG isotype control (Invitrogen) for 30 min at 4 °C. The cells were then incubated with secondary goat anti-rabbit AffiniPure F(ab’)_2_ IgG Alexa-488 (Jackson ImmunoResearch) for 30 min at 4 °C. Samples were analyzed using the BD FACSCanto™ flow cytometer (BD Biosciences) and Kaluza 2.1 Software (Beckman Coulter).

### Actin polymerization assay

PBMCs were incubated with or without N-formyl-methionyl-leucyl-phenylalanine (fMLP) (Sigma-Aldrich, USA) for 20 seconds and immediately fixed with 50 μL of 4% formaldehyde and incubated for 25 min at room temperature. The cells then were washed in PBS and stained with CD14 PE (BD Biosciences, CA, USA, catalog number 345785), permeabilized using BD Perm/Wash Buffer (BD Biosciences, CA, USA), and stained with Phalloidin Alexa Flour 647 (Invitrogen, Thermo Fisher Scientific, USA, catalog number A22287) (14). After incubation, the cells were analyzed using a FACS Canto II (BD Bioscience, CA, USA). Data analysis was performed using Kaluza 2.1 Software (Beckman Coulter Life Sciences, USA). The increase in mean fluorescence intensity (MFI) in stimulated samples was calculated as (stimulated samples/unstimulated sample) * 100.

## Data Availability

All relevant data is contained within the article: the original contributions presented in the study are included in the article, further inquiries can be directed to the corresponding author.
